# Evaluating Hybridization Chain Reaction to Improve miRNA Measurements at Portable Electroanalytical Strip: miRNA-21 as a Case of Study

**DOI:** 10.1021/acsomega.5c09666

**Published:** 2026-02-09

**Authors:** Ada Raucci, Assunta Anna Santillo, Luca Capelli, Antonio Giordano, Ibrahim A. Darwish, Alessandro Bertucci, Stefano Cinti

**Affiliations:** † Department of Pharmacy, 9307 University of Naples Federico II, 80131 Naples, Italy; ‡ Department of Breast and Thoracic Oncology, Istituto Nazionale Tumori IRCCS Fondazione G. Pascale, Napoli 80131, Italy; § Department of Chemistry, Life Sciences and Environmental Sustainability, 9370 University of Parma, Parma 43124, Italy; ∥ Sbarro Institute for Cancer Research and Molecular Medicine, Center for Biotechnology, College of Science and Technology, 6558 Temple University, Philadelphia, Pennsylvania 19122, United States; ⊥ Department of Medical Biotechnologies, University of Siena, 53100 Siena, Italy; # Department of Pharmaceutical Chemistry College of Pharmacy, King Saud University, P.O. Box 2457, Riyadh 11451, Saudi Arabia; ∇ Department of Chemistry, Faculty of Science, Chulalongkorn University, Bangkok 10330, Thailand

## Abstract

This work reports on the evaluation of the hybridization chain reaction recognition system to be combined with a frugal sensing platform, namely screen-printed electrode, for the measurement and the amplification of circulating nucleic acids, without the use of time-consuming and complex procedures. In fact, if traditional strategies usually rely on nanomaterials or intricate modifications, our method places the target sequence and hairpins directly on the electrode surface, reducing both cost and preparation time while maintaining efficient signal amplification. Two specific DNA-based hairpins, modified with methylene blue as a redox mediator, has been rationally designed and characterized, yielding a “signal-off” response triggered by the presence of miRNA target. The system has been applied toward both standard and human serum samples, obtaining satisfactory detection limit of ca. 100 pM, with a repeatability less than 10%. This platform does not require modification and/or complex work flow, it offers a cost-effective, sensitive, and decentralized solution for point-of-care diagnostics and real-time miRNA monitoring in clinical settings, not only for cancer monitoring.

## Graphical abstract

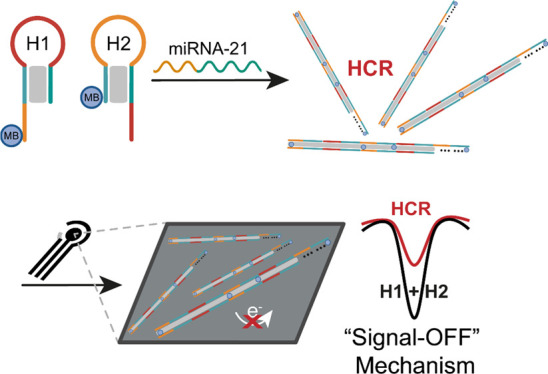

## Introduction

MicroRNAs (miRNAs) are small noncoding RNA molecules that play crucial roles in gene expression and metabolic functions.^1^ They have garnered considerable attention for their ability to modulate cellular mechanisms, influencing cell proliferation, angiogenesis, cancer metastasis and drug resistance.^2–5^ Among the various miRNAs, miRNA-21 has emerged as one of the most widely studied due to its prominent role in oncogenesis and other pathological conditions.^6^ Given their pivotal role in important cellular processes, miRNAs, including miRNA-21, are considered valuable indicators of disease progression and promising targets for treatment strategies.^7–10^ Therefore, detection and quantification of miRNAs in diseases are essential to understand their role in biological processes and their potential as biomarkers.

Several techniques have been developed for the detection of miRNAs, each with its own advantages and limitations, including Northern blotting,^11^ quantitative real-time PCR (qRT-PCR),^12^ and microarrays.^13^ While these methods offer high sensitivity and specificity, they also involve higher cost and complexity, limitings their widespread application. For example, PCR-based detection requires short primers that reduce efficiency and increase the risk of nonspecific amplification. Additionally, the low concentration of miRNA in cells and blood also limits the sensitivity of Northern blotting, making it inadequate for detecting nanomolar or even lower concentrations. Despite their high throughput, microarrays suffer from cross-hybridization and low sensitivity.^14,15^ To address these challenges, innovative techniques such as colorimetric,^16^ fluorescent^17^ and electrochemical methods are being developed for miRNA detection.^18^ Although, many of these techniques utilize cutting-edge technology, they often necessitate multiple reactions or signal transformation steps. Here, we addressed these biosensing challenges by employing electrochemical strips as promising biosensors offering low cost, immediacy, miniaturization and rapid response.^19–21^ However, a challenge in designing electrochemical biosensors is precisely controlling the density and orientation of the recognition probes on the electrode surface, as these parameters are critical for defining their response window and sensitivity.^22–24^

To tackle these challenges and further improve the sensitivity of electrochemical biosensors, several chemical and biological amplification strategies have been proposed.^25–27^ Among these, the hybridization chain reaction (HCR) has emerged as a promising technique. HCR is a DNA polymerization cascade triggered by initiator or target molecules, leading to the formation of long DNA chains from short oligonucleotide hairpins.^28,29^ This isothermal amplification method leverages the self-assembly of two rationally designed DNA hairpins to produce amplified signals in the presence of a target molecule.^30,31^ When a trigger strand initiates the chain reaction, it consecutively opens the hairpin structures, causing them to hybridize in a self-sustained process and form long DNA polymers, significantly enhancing the detection signal and enabling the identification of target molecules at very low concentrations.^32,33^ Compared to other amplification techniques, HCR offers superior advantages, such as low background noise, cost-effectiveness, and greater stability, making it a powerful tool for developing sensitive biosensors.^34,35^

Several electrochemical methods for miRNA detection harnessing HCR amplification have been reported.^36–41^ However, these methods often require the use of specific nanomaterials or involve labor-intensive and time-consuming electrode modification procedures. To address these limitations, in the present work, we developed an innovative platform for the detection of miRNA-21 that integrates a cost-effective polyester electrode combined with HCR amplification. The use of HCR as signal amplification strategy enhances sensitivity and greatly simplifies the manufacturing process, achieving a detection limit at the picomolar level without the need for electrode surface modification. By eliminating complex surface functionalization procedures, this advancement facilitates fabrication and reduces inconsistencies. Various influential miRNA-21 sensors achieve ultralow detection limits (LODs) by relying on engineered, immobilized interfaces: tetrahedral DNA scaffolds on modified gold surface,^36^ hierarchical/“flower-like” gold nanostructures,^42^ or immobilized hairpins followed by target-triggered HCR.^38,40^ These formats are powerful but require multistep surface chemistry (capture-layer assembly, nanostructure growth, blocking), which raises cost, fabrication time, and batch-to-batch variability. Homogeneous, immobilization-free HCR concepts have been explored at bench scale,^41^ yet they are typically demonstrated on conventional electrodes rather than printed, portable strips. In our approach, the sample is applied directly to the electrode surface, significantly lowering both the overall cost and time required for analysis. The DNA hairpins used in the sensor were modified with methylene blue (MB) as a redox mediator. The HCR technique enables signal amplification through a signal-off mechanism, where electron transfer is substantially reduced when the redox mediators become intercalated within the self-assembled DNA polymers, thereby enabling highly sensitive miRNA detection. This proposed platform leverages the principles of the hybridization chain reaction for the sensitive detection of miRNA-21, offers a robust, cost-effective system with great potential for point-of-care (PoC) diagnostics and real-time monitoring of miRNA levels in clinical settings.

## Experimental Section

### Reagents and Apparatus

All reagents used were of the highest quality available. PBS tablets (140 mM NaCl, 10 mM phosphate buffer, 3 mM KCl), and human serum were purchased from Sigma-Aldrich (St. Louis, MO, USA). The DNA hairpin probes, specifically hairpin H1 (5′-Atto MB2-ATC AGT CTG ATA AGC TAC TAA CTT AGC TTA TCA G-3′) and hairpin H2 (5′-Atto MB2-TAG CTT ATC AGA CTG ATC TGA TAA GCT AAG TTA G-3′), along with the target sequence miRNA-21 (5′-uag cuu auc aga cug aug uug-3′), were obtained from Metabion GmbH (Steinkirchen, Germany). Sequences tested as potential interferents, including miRNA-218 (5′-uug ugc auc uaa cca ugu-3′), miRNA-29c-5p (5′-uga ccg auu ucu ccu ggu guu-3′), and miR652-5p (5′-caa ccc uag gag ggg gug cca uuc-3′), miRNA-101-5p (5′-cag uua uca cag ugc uga ugc u-3′), miRNA-107 (5′-agcagcauuguacagggcuauca-3′), anti-miR-155 LNA 50% B-LNA Oligo (5′-(+A)­(+C)­(+C)­(+C)­(+C)­(+T)­(+A)­(+T)­(+C)­(+A)­(+C)­G ATT AGC ATT AA-3′), the longer single-stranded DNA oligonucleotide (5′-CTA AAG ACC ATT GCA CTT CGT GCC CGA AAC GCC GAA CAC AAT CCC AAG CGG TTT GCT GCG GTA ATC ATG AGG ATA AGA GAG CCA CGA ACC-3′), were also sourced from Metabion GmbH (Steinkirchen, Germany). Adobe Illustrator was used to draw the wax model of the creation of the hydrophilic test area on the filter- paper electrodes. A solid ink printer, the ColorQube 8580 from Xerox (USA), was used to print the hydrophobic wax layer.

### Fabrication of Electrochemical Strips

Graphite-based polyester screen-printed electrodes (SPE) with a three-electrode configuration were produced in-house by manual screen printing onto a flexible polyester film substrate, Autostat HT5 (125 μm), MacDermid, U.K. The three-electrode design was created manually using a squeegee to distribute the conductive inks through a specially designed mask. Specifically, Ag/AgCl ink (Loctite, Italy) was used to print the connections and reference electrode, while carbon ink (Sun Chemical, USA) was used for the working and contrast electrodes. After printing, the strips were thermally cured at 100 °C for 30 min, making the ink stable for electrochemical measurements. The diameter of the working electrode was 0.4 cm, while the electrochemical strips measured approximately 2.5 cm high and 1 cm wide. To prevent the spread of aqueous samples toward the connector, adhesive tape was applied to delimit the test area. SPEs on flexible/polyester substrates stored dry in sealed pouches at room temperature have been reported to retain a reliable electrochemical response for several months, in line with our storage conditions.^43,44^ Regarding the fabrication of the filter paper electrodes, before printing the silver and graphite layer, we followed the process illustrated below: the final model of the paper device was created using Adobe Illustrator and then printed on Whatman No. 1 chromatographic paper with wax-based ink. After printing, the wax paper was placed in an oven at 100 °C for 1 min. This procedure allowed the wax to penetrate through the paper, forming a hydrophobic layer around the hydrophilic test area. This hydrophobic layer is crucial for clearly defining the test area and limiting the diffusion of the solution within the electrochemical cell.^45^

### Electrochemical Measurements

All electrochemical measurements were performed with a Multi Emstat 4 portable potentiostat (PalmSens, Netherlands) equipped with a multi-8 reader and interfaced with a laptop running PSTrace 5.9 software. All reported potentials refer to the Ag/AgCl reference pseudoelectrode of the screen-printed electrochemical strips. Square wave voltammetry (SWV) was used to analyze the biosensor surfaces with the samples. SWV voltammograms were collected using a frequency of 50 Hz, an equilibrium time of 5 s, an E step of 0.001 V, an amplitude of 0.01 V and a potential window between 0 and −0.5 V, as previously optimized.^46^

### HCR-Amplified Electrochemical miRNA Detection

The buffer used for the electrochemical measurements and preparation of the H1, H2 and miRNA-21 solutions was a PBS buffer at pH 7.4, containing 140 mM NaCl, 10 mM phosphate and 3 mM KCl.

Regarding the experimental procedure, DNA hairpin solutions, H1 and H2, both modified with MB as a redox mediator, were mixed together to a desired final concentration and then incubated in an Eppendorf for 30 min, both in the absence and in the presence of different concentrations of miRNA-21. After the incubation time had elapsed, a volume of 50 μL was taken and placed directly on the working area of the electrode. The current intensity was measured for samples containing only H1 and H2 compared to those containing different miRNA-21 concentrations. For all measurements, the signal variation (%) was evaluated as signal variation (%) = (*I*_0_ – *I*_target_)/*I*_0_ × 100, where *I*_0_ is the signal obtained in the absence of miRNA and *I*_target_ is the signal obtained in the presence of miRNA. Because our readout is signal-off (HCR sequesters MB away from the electrode), signal change (%) is positive and increases with target concentration. We chose relative percentage change rather than absolute change because SPEs inherently exhibit differences from strip to strip; normalization to *I*_0_ suppresses these multiplicative factors and allows for a fair comparison between substrates and matrices (polyester vs paper; buffer vs diluted serum). The same procedure was performed for both standard and human serum measurements.

## Results and Discussion

### Evaluation of the Experimental Setup

Optimization of the experimental parameters for the electrochemical biosensor was performed in a standard phosphate buffer solution at pH 7.4 to ensure optimal analytical performance in target detection. Measurements were conducted simultaneously on eight electrodes using an eight-channel multiplexer potentiostat.

As previously mentioned, the two DNA hairpins, H1 and H2, were designed to remain in a closed conformation in the absence of miRNA-21 (Figure S1A,B in the Supporting Information (S.I.)), thus maintaining a stable electrochemical signal due to the MB tags conjugated at their 5′-end. MB is known to intercalate into double-stranded DNA (dsDNA), as demonstrated in previous studies.^47,48^ In our system, this property provides a plausible explanation for the observed signal-off effect. When the DNA is in a hairpin conformation, MB molecules remain relatively free and accessible for electron transfer. Upon HCR polymers formation, however, extended dsDNA structures are formed that allow MB intercalation. This intercalation increases the distance of MB molecules from the electrode surface and introduces steric hindrance, both of which reduce electron transfer efficiency, leading to a decrease in the electrochemical signal and enabling a signal-off mechanism. (Scheme 1). Such reductions in electrochemical response from intercalated MB have been consistently reported in prior studies.^49–51^

**Scheme 1:**
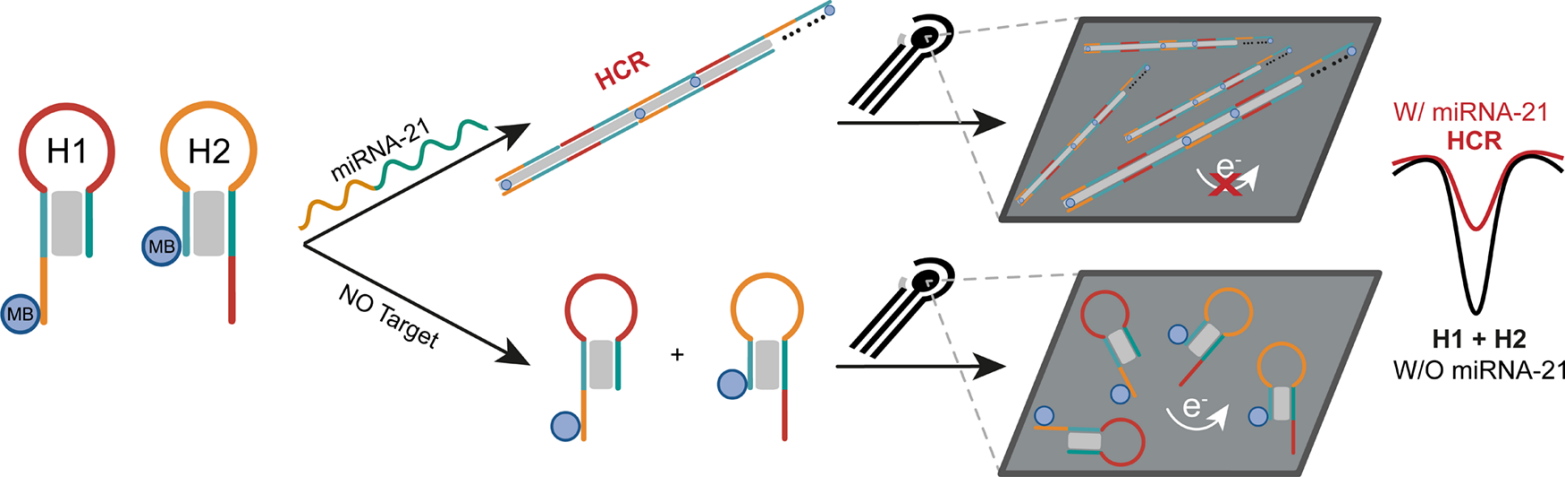
Schematic Representation Illustrating the Proposed HCR-Based Signal-Off Mechanism for the Electrochemical Detection of miRNA-21

This signal-off mechanism offers high sensitivity, as even small amounts of miRNA-21 can cause a significant reduction in the electrochemical signal due to the amplification effect provided by HCR. The extent of the signal decrease is directly proportional to the concentration of the target miRNA-21, enabling accurate and highly sensitive quantification of miRNA.

The initial optimization phase focused on selecting the optimal low-cost substrate for the screen-printed carbon electrodes, comparing polyester and chromatographic paper as shown in Figure 1A.

**Figure 1:**
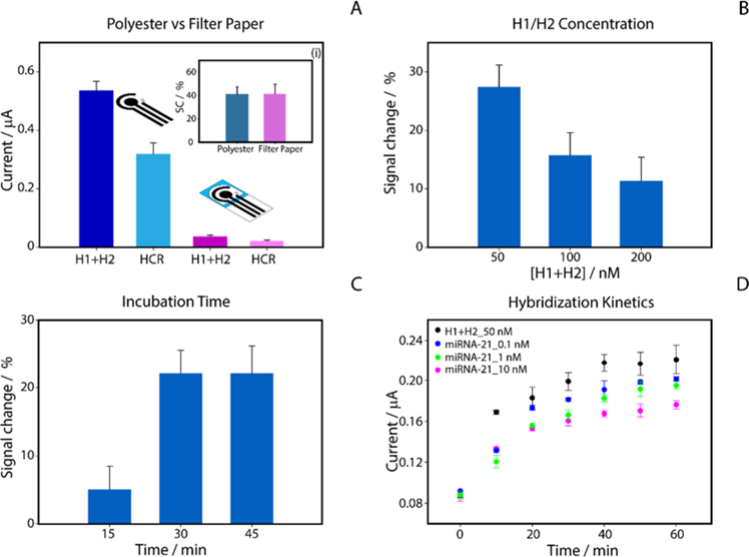
Optimization of the experimental parameters for the miRNA-21 detection. (A) Comparison of signal response on polyester (blue) and chromatographic paper (pink) substrates at 100 nM miRNA-21 concentration. Inset (i): Histogram showing signal change (%) in the presence of miRNA-21 on a polyester-based substrate (blue) and filter paper (pink). (B) Optimization of H1+H2 concentrations (50, 100, and 200 nM); (C) evaluation of incubation times (15, 30, and 45 min); (D) hybridization kinetics between H1 and H2 at 50 nM and varying miRNA-21 concentrations (0.1, 1, and 10 nM) over 60 min.

Despite a similar relative signal change % was observed when using a concentration of 100 nM miRNA-21, the polyester substrate was selected for its robustness and ability to generate a higher absolute current signal from the presence of MB.

In the context of HCR for miRNA-21 detection, the concentration of the two hairpin probes, H1 and H2, plays a crucial role in determining the resulting electrochemical signal. In order to maximize the signal change %, we tested different concentrations of H1+H2 at 50, 100, and 200 nM, keeping the concentration of miRNA-21 constant at 100 nM (Figure 1B). In all HCR assays, we employed equimolar concentrations of the two hairpins. This was done because HCR proceeds through the alternate opening of both structures, so an imbalance would make one of them limiting and slow the reaction, while excess MB-labeled hairpin would increase the background signal and reduce the percentage signal change. Consequently, another key aspect was the optimization of the incubation time required for the HCR process. We first incubated H1+H2 at a concentration of 50 nM, both in the absence and presence of 100 nM miRNA-21, allowing the reaction to proceed for 15, 30, and 45 min, as shown in Figure 1C. Next, we evaluated the signal decrease rates of the H1+H2 solutions in the presence of the target miRNA-21 compared to the signal obtained with H1+H2 alone. The results showed that a reaction time of 30 min led to a 22% signal decrease, compared to only 5% decrease with 15 min incubation. No significant difference was observed between 30 and 45 min. Therefore, we selected 30 min as the optimal incubation time for our analysis. Next, we studied the concentration dependence of the hybridization kinetics, testing H1 and H2 at a concentration of 50 nM either alone or in the presence of miRNA-21 at concentrations of 0.1, 1, and 10 nM. As shown in Figure 1D, a 30 min incubation was sufficient to achieve both sensitivity and repeatability, as extending the time beyond 30 min did not significantly improve detection of different concentrations. In our assay, the HCR is performed in solution and only the final mixture is deposited on the bare SPE. H1/H2 dried on paper and stored up to 14 days were redissolved and measured, showing comparable current to day 0 in the first days and a decrease only at longer times (Figure S2, SI). In a PoC perspective, the overall time-to-result can be estimated as follows: sample preparation/dilution ≈5–10 min, HCR incubation 30 min, and electrochemical measurement <5 min, leading to a total assay time of about 40 min.

### Analytical Characterization in Standard and Human Serum

After reviewing all key experimental features, we evaluated the analytical performance of the printed platform for HCR-based miRNA-21 detection. Initially, we conducted an evaluation in buffer solution by examining increasing concentrations of the target from 0.01 to 500 nM, as illustrated in Figure 2.

**Figure 2:**
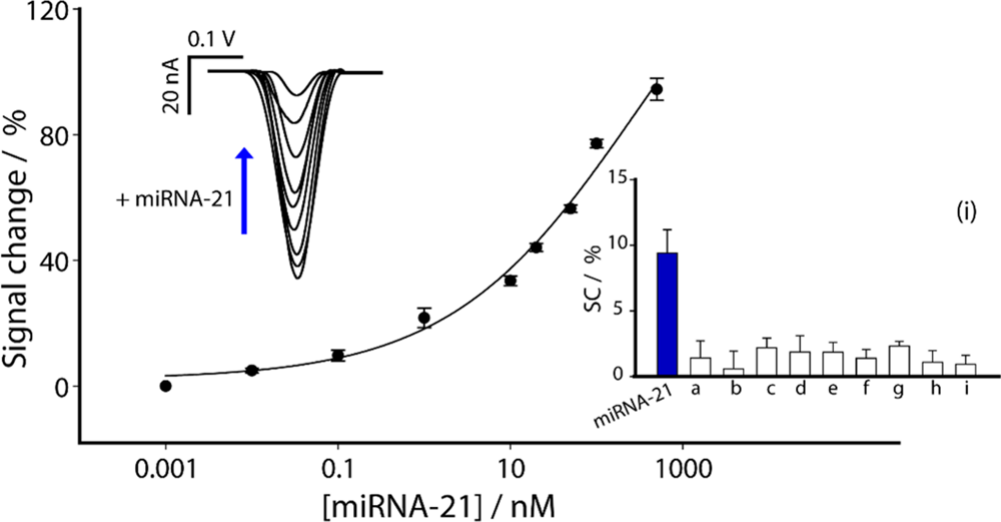
Calibration curve and SWV curves obtained in buffer solution by testing different concentrations of miRNA-21 target from 0.01 to 500 nM. Selectivity studies are reported in the inset (i) comparing the signal intensities obtained in the presence of not-complementary miRNAs (a–e), high-affinity LNA-modified antimiR (f), a longer single-stranded DNA oligonucleotide (g), and short double-stranded DNA oligonucleotides (h,i). All the experiments have been carried out in triplicate. SWV parameters: t eq = 5 s, E start = 0.0 V, E end = – 0.5 V, E step = 0.001 V, Amplitude = 0.01 V, Frequency = 50.0 Hz.

Optimized settings were applied for all experiments, revealing a semilogarithmic sigmoidal relationship between signal change and target concentration (expressed on a logarithmic scale). It is important to highlight that the percentage signal change shown in the graphs reflects a reduction in the signal, as the system operates in a signal-off mode. The correlations were satisfactory, with a coefficient of determination *R* ^2^ of 0.993. Furthermore, the LOD was calculated based on the miRNA-21 concentration that produced a 10% change in the electrochemical signal relative to the baseline and resulted to be approximately at 100 pM. First, the baseline signal was recorded in the absence of miRNA-21. Then, a series of miRNA-21 concentrations (ranging from 0.01 to 500 nM) was tested, and the corresponding electrochemical responses were measured. The LOD was defined as the lowest miRNA-21 concentration at which the signal reached 10% of the maximum response observed in the saturation region. This practical approach was chosen because a 10% signal change is sufficiently large to be reliably distinguished from background noise yet still reflects the sensor’s response at low target concentrations. As previous discussed,^52^ defining the LOD based on a significant fraction (e.g., 10%) of the maximum signal ensures a robust and reproducible estimation of the sensor’s sensitivity, minimizing the risk of artifacts arising from background fluctuations. The repeatability, reported as the relative standard deviation of the response, the percent signal change, as RSD% = 100 × SD/mean, was good, with a coefficient of variation of 2% (calculated on five replicates at a target concentration of 50 nM). The results are highly promising, suggesting that the proposed HCR-based miRNA-21 detection platform offers significant potential for clinical application, particularly in terms of ease of use, short production time, high sensitivity and low cost. The platform’s selectivity was also evaluated using an extended panel of interferents (miRNA-218, miRNA-29c-5p, miRNA-652-5p, miRNA-101, and miRNA-107), an LNA-modified antimiR-155, a longer single-stranded DNA oligonucleotide and short double-stranded DNA oligonucleotides, each at a concentration of 0.1 nM. As shown in the inset of Figure 2, at 0.1 nM, noncomplementary miRNAs produced signal changes ≤2%, whereas miRNA-21 gave a response of about 10%, indicating a clearly higher response for the target sequence. This behavior is consistent with the solution-phase HCR specificity observed by fluorescence and gel analysis, where three random RNA sequences did not activate the reaction (Figure S3, SI).

After examining the platform’s performance in model conditions, we extended our study to a complex biological matrix such as human serum. During the first tests with undiluted serum, we observed a significant matrix effect, which compromised effective reading of the electrochemical signal. This prompted us to conduct a thorough evaluation of the matrix effect, as illustrated in Figure 3A.

**Figure 3:**
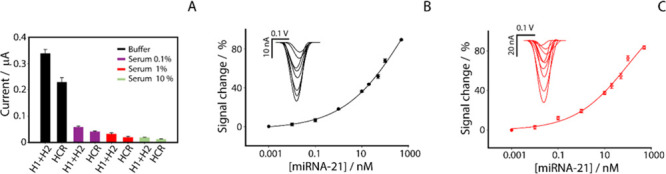
Characterization of electrochemical sensor performance for miRNA-21 detection in buffer and human serum. (A) Study of the matrix effect in the presence of 50 nM miRNA-21 in buffer (red) and human serum diluted to 0.1% (purple), 1% (orange), and 10% (green), before and after introduction of the miRNA-21 target. All bars were obtained as an average of three replicates; calibration and SWV curves obtained in (B) phosphate buffer using a H1/H2 concentration of 100 nM; (C) 1% diluted serum using a H1/H2 concentration of 100 nM. All experiments were performed in triplicate, and the experimental conditions are as shown in the caption of Figure 2.

To address this challenge, we decided to explore modifying the optimized concentration in buffer, and we tested the sensor using a H1 and H2 concentration of 100 nM together with a miRNA-21 concentration of 50 nM, in the presence of different serum dilutions (10, 1, 0.1%). The increase in H1 and H2 concentrations was thought to facilitate the detection of the electrochemical signal, counterbalancing the suppression effect observed in serum. As the graph in Figure 3 shows, even in the absence of the target, the absolute current value with only H1+H2 is significantly lower than that observed in buffer. However, the relative signal decrease percentages in the presence of miRNA-21 for all serum dilutions tested were similar to those achieved in buffer, being around 35%. These results indicate that, despite a significant matrix effect caused by human serum on the measurable currents, the sensor retains a good ability to discriminate and detect miRNA-21, exhibiting a consistent signal-off effect even under complex biological matrix conditions. This result is of crucial importance for the potential deployment of the platform in clinical settings, where the ability to function effectively in the presence of biological matrices such as human serum is essential.

Next, we explored the performance of the sensor by testing it at different concentrations of miRNA-21, varying the concentrations of DNA hairpins H1 and H2 at 50 and 100 nM, and evaluating the performance in both phosphate buffer and serum diluted to 0.1 and 1%. As previously described, the calibration curve obtained in buffer using H1+H2 at a concentration of 50 nM demonstrated the ability to detect miRNA-21 up to 100 pM. In contrast, Figure 3B shows the calibration curve obtained in buffer with H1+H2 at a concentration of 100 nM. Under these conditions, the correlation yielded an *R* ^2^ value of 0.998, with a LOD set at 160 pM and a repeatability of 4%, calculated at a target concentration of 50 nM. In terms of quantification capability, the platform exhibited a linear range between 10 and 100 nM in buffer, described by the regression equation *y* = 0.333*x* + 34.75 (*R* ^2^ = 0.97), Figure S4A, SI. To mitigate the matrix effect caused by serum, Figures 3C presents the calibration curve obtained in 1% serum, using H1+H2 at a concentration of 100 nM, obtaining a comparable linear range of 10–100 nM, with regression *y* = 0.372*x* + 35.85, Figure S4B, SI. The correlation was satisfactory, with determination coefficient of *R* ^2^ = 0.993, and a low limit of detection of 90 pM. Accuracy at the 50 nM spike, yielded a mean of 46.2 nM, corresponding to 92.4% accuracy, which falls within common bioanalytical acceptance ranges and supports the reliable and reproducible quantification of miRNA-21 in serum. Repeatability, calculated from five replicates at a target concentration of 50 nM, resulted to be ca. 5%. However, we also tested the performance of the miRNA sensing in plasma samples, obtaining similar results in terms of analytical performance, as illustrated in the Table S1 in the SI.

## Conclusions

In conclusion, this study developed and evaluated a highly sensitive platform for miRNA-21 detection, utilizing an electrochemical biosensor integrated with the hybridization chain reaction (HCR) on a polyester electrode. By eliminating the need for complex surface modifications, the platform significantly simplified the fabrication process, enhancing reproducibility and reducing production costs. The system demonstrated excellent sensitivity, with a detection limit in the picomolar range, and strong specificity, as evidenced by minimal interference from other closely related miRNAs. Moreover, the platform’s robustness was validated in complex biological matrices, such as human serum, further highlighting its potential for clinical applications in disease diagnosis and monitoring. The results indicate that this biosensing approach is well-suited for point-of-care (PoC) diagnostics, offering a reliable, cost-effective tool for real-time miRNA analysis. Overall, this work not only advances electrochemical biosensor technology for miRNA detection but also paves the way for its practical use in clinical settings. It holds promise for enabling more precise and personalized disease management based on miRNA profiling, ultimately contributing to improved diagnostic and therapeutic strategies.

## Supplementary Material



## Notes

Fluorescence kinetic profiles for the formation of HCR polymers, stability study of H1/H2 hairpins dried on paper, fluorescence kinetic profiles for the specificity test of HCR reaction, and regression line corresponding to the linear range of the calibration curves for miRNA-21 (PDF)
