# Supplementary material for: Evaluating Hybridization Chain Reaction to Improve miRNA Measurements at Portable Electroanalytical Strip: miRNA-21 as a Case of Study
Source: ACS Omega. 2026 Feb 9;11(7):11578–84. doi: 10.1021/acsomega.5c09666 (PMC12947044; doi:10.1021/acsomega.5c09666)
Supplement: Supplementary file 1 [file ao5c09666_si_001.pdf]

Supporting Information

Evaluating Hybridization Chain Reaction to Improve miRNA Measurements at Portable Electroanalytical Strip: miRNA-21 as the case of Study

Ada Raucci [1,2], Assunta Anna Santillo [1], Luca Capelli [3], Antonio Giordano [4,5], Ibrahim A. Darwish[6], Alessandro Bertucci [3*], Stefano Cinti [1,4,7*]

[1] Department of Pharmacy, University of Naples Federico II, Naples 80131, Italy

[2] Department of Breast and Thoracic Oncology, Istituto Nazionale Tumori IRCCS Fondazione G. Pascale, Napoli 80131, Italy

[3] Department of Chemistry, Life Sciences and Environmental Sustainability, University of Parma, Parma 43124, Italy

[4] Sbarro Institute for Cancer Research and Molecular Medicine, Center for Biotechnology, College of Science and Technology, Temple University, Philadelphia, PA 19122, USA

[5] Department of Medical Biotechnologies, University of Siena, 53100 Siena, Italy.

[6] Department of Pharmaceutical Chemistry College of Pharmacy, King Saud University, P.O. Box 2457, Riyadh 11451, Saudi Arabia

[7] Department of Chemistry, Faculty of Science, Chulalongkorn University, Bangkok 10330, Thailand.

Corresponding authors: Alessandro.bertucci@unipr.it; Stefano.cinti@unina.it

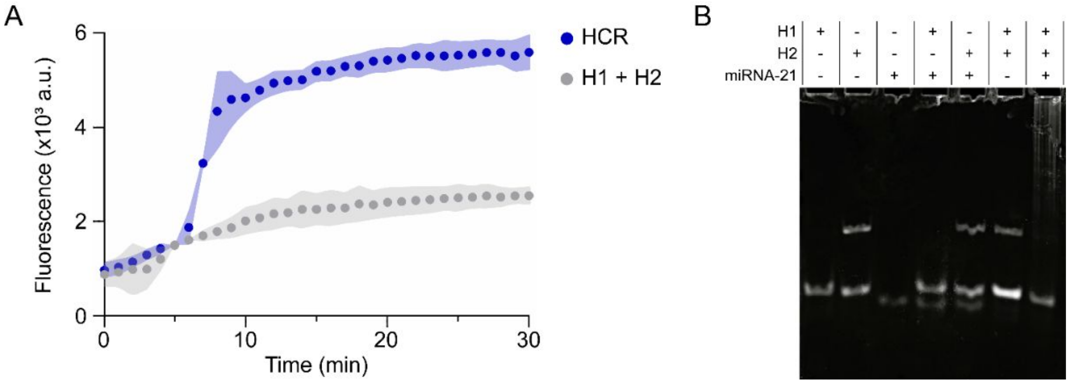

Figure S1. A) Fluorescence kinetic profiles for the formation of HCR polymers ([H1] = [H2] = 100 nM; [miRNA] = 10 nM. n = 3, mean ± SD). B) Native 10% acrylamide gel electrophoresis showing the formation of HCR polymers. Lane 1: H1 (100 nM); Lane 2: H2 (100 nM); Lane 3: miRNA21 (10 nM); Lane 4: H1 (100 nM) + miRNA-21 (10 nM); Lane 5: H2 (100 nM) + miRNA-21 (10 nM); Lane 6: H1 (100nM) + H2 (100nM); Lane 7: H1 (100nM) + H2 (100nM) + miRNA-21 (10 nM).

To carry out fluorescence experiment we used a H2 hairpin presenting a fluorophore-quencher pair: H2-FQ: 5’-(BHQ-1)-TAG CTT ATC AGA CTG ATC TGA TAA GC(6-Fam-dT) AAG TTA G-3’.

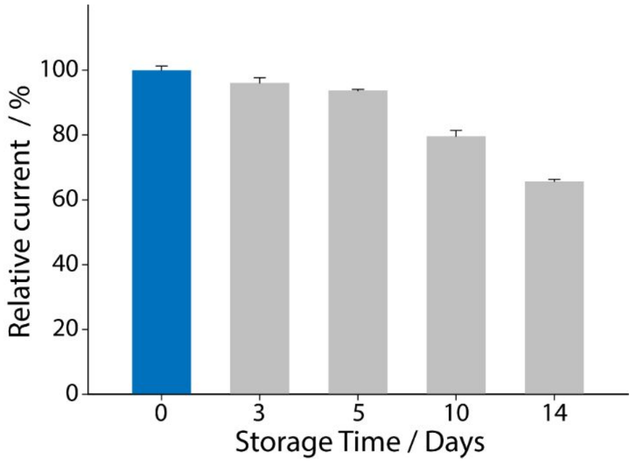

Figure S2. Stability study of H1/H2 hairpins dried on paper. Histogram showing the relative current (%) recorded at day 0, 3, 5, 10 and 14 after re-dissolution and electrochemical measurement. Relative current was calculated as (I_x / I_0) × 100, where I_0 is the signal at day 0.

	Sequence
Interfering RNA 1	AAAAGCCGCUAGGCCGUUGCCUAG
Interfering RNA 2	UAAUUUCUACUAAGUGUAGA
Interfering RNA 3	UGAGGUAGUAGGUUGUAUAGUU

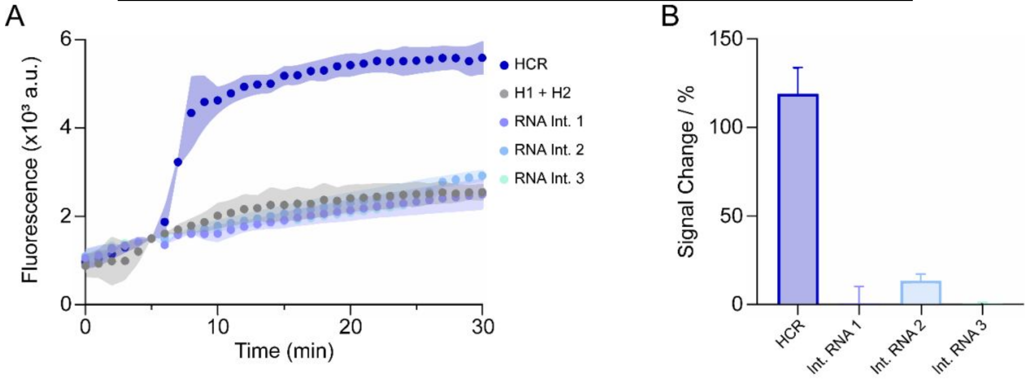

Figure S3. A) Fluorescence kinetic profiles for the specificity test of HCR reaction ([H1] = [H2] = 100 nM; [miRNA] = [Interfering RNA] = 10 nM. n = 3, mean ± SD). B) Signal Change % relative to specificity test. Calculated at minute 30 (n = 3, mean ± SD).

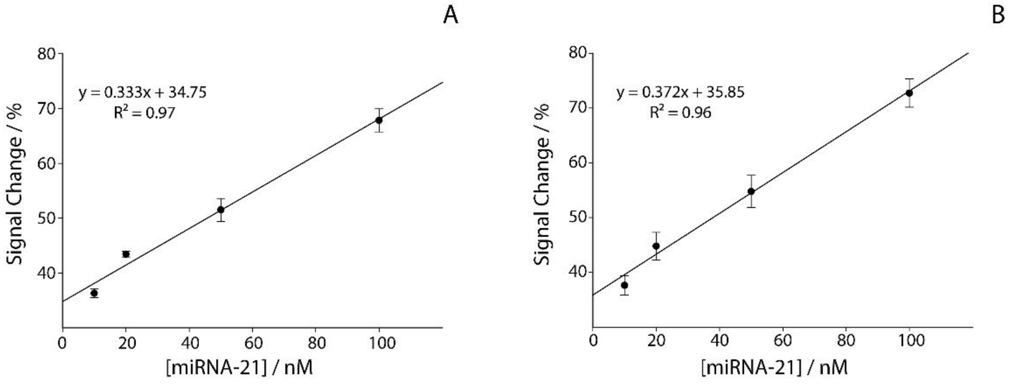

Figure S4. Regression line corresponding to the linear range of the calibration curves for miRNA-21. (A) Signal change (%) vs miRNA-21 concentration in buffer, with the regression line in the 10–100 nM range and the corresponding linear equation. (B) Signal change (%) vs miRNA-21 concentration in diluted human serum (0.1%), with the regression line in the 10–100 nM range and the corresponding linear equation.

Table S1. Comparison of the performance of the miRNA sensing in serum and plasma samples.

Matrix	Equation	LOD (pM)	CV (%)
Serum	y = 0.82 + (133.05·x^0.46)/(112.44^0.46+x^0.46)	90	5
Plasma	y = 6.65 + (70.75·x^0.88)/(23.60^0.88 + x^0.88)	70	4
